# Validation of the PECARN clinical decision rule for children with minor head trauma: a French multicenter prospective study

**DOI:** 10.1186/s13049-016-0287-3

**Published:** 2016-08-04

**Authors:** F. Lorton, C. Poullaouec, E. Legallais, J. Simon-Pimmel, M. A. Chêne, H. Leroy, M. Roy, E. Launay, C. Gras-Le Guen

**Affiliations:** 1 Department of Pediatric Emergency, University Hospital, Quai Moncousu 44093 Nantes Cédex 01, France; 2 INSERM CIC 1413, University Hospital, 38 bd Jean Monnet, 44093 Nantes Cédex 01, France; 3 Department of Pediatrics, Hospital of Saint-Nazaire, 11 bd Georges Charpak, 44 606 Saint-Nazaire Cédex, France; 4 Department of Emergency, Departmental Hospital of Vendée, Les Oudairies, 85925 La Roche sur Yon Cédex 9, France; 5 Department of Pediatrics, University Hospital, Quai Moncousu 44093 Nantes Cédex 01, France

**Keywords:** Minor head trauma, Children, External validation, Clinical decision rule, Cranial computed tomography

## Abstract

**Background:**

To date, the Pediatric Emergency Care Applied Research Network (PECARN) rule for identifying children who are at very low risk of clinically-important traumatic brain injuries after minor head trauma has not been validated prospectively in an independent population. Our goal was to evaluate the diagnostic performance of the PECARN clinical decision rule in a French pediatric population in multiple clinical settings.

**Methods:**

We conducted a multicenter, prospective, non-interventional cohort study of patients with minor head trauma who presented to three emergency departments in France. We enrolled patients younger than 16 years of age seeking a consultation within 24 h of head trauma with Glasgow Coma Scale scores of 14–15.

**Results:**

During the study period, we included 1499 children of which 421 (28 %) were under 2 years of age, and 955 (64 %) were male. A cranial computed tomography (CT) scan was performed on 76 patients (5.1 %). Of the 1499 included patients, 9 children (0.6 %) had a clinically-important traumatic brain injury, and none were classified as very low risk by the PECARN rule. In our study, the sensitivity of this clinical decision rule was 100 % (95 % CI 66.4 to 100 %), the specificity was 69.9 % (95 % CI 67.5 to 72.2 %) and the negative predictive value was 100 % (95 % CI 99.7 to 100 %).

**Discussion:**

Our study confirmed the good predictive performances of the PECARN clinical decision rule for minor head trauma in children. The PECARN rule performed similarly to our study and to its internal validation study.

**Conclusions:**

We conducted an external validation study of the PECARN clinical decision rule for the detection of clinically-important traumatic brain injuries in children with minor head trauma, according to the methodological standards. The PECARN rule successfully identified all patients with clinically-important traumatic brain injuries, with a limited use of CT scans. Conducting a broad validation study with a large cohort is a prerequisite to provide sufficient statistical power before authorizing its implementation and generalization.

**Trial registration:**

This study has been registered in ClinicalTrials.gov with identifier number: NCT02752711 on April 27, 2016.

## Background

Head injuries in children are a common cause for emergency department visits. More than 95 % of these constitute minor head trauma (MHT), defined as Glasgow Coma Scale (GCS) score greater than or equal to 13. Among these patients, less than 10 % have traumatic brain injuries (TBI) and less than 1 % need neurosurgery [1–4].

A cranial computed tomography (CT) scan is the diagnostic standard for identifying the presence of TBI, and its use has tripled in the USA between 1995 and 2008 [5]. This leads to potentially unnecessary exposure of children to ionizing radiations, which carry associated risks of leukemia and brain tumors, especially in children under 10 years of age [6, 7]. Physicians are faced, however, with a diagnostic dilemma, as TBIs need to be identified rapidly.

Clinical decision rules (CDR) may prove useful for helping physicians to identify children with a significant risk of TBI. CDR’s are clinical tools for improving accuracy in medical decision-making, and for minimizing the use of potentially harmful diagnostic tests. The Pediatric Emergency Care Applied Research Network (PECARN) rule for children with MHT was considered to have the best methodological quality among pediatric rules [8, 9]. This rule was derived and then validated in 42,412 patients, and it aimed to identify children with a very low risk of clinically-important TBI (ciTBI) for which the cranial CT may safely be avoided [2]. The CDR had a sensitivity for the presence of ciTBI of 100 %, 95 % CI 86.3–100, for children younger than 2 years of age, and of 96.8 %, 95 % CI 89–99.6, for children aged 2 years and older.

Before being recommended for use in routine practice, a CDR must be rigorously developed, validated, and implemented. Methodological standards for the development of CDRs have been described previously [10–13] and they include several steps: i) creating the rule (derivation), ii) testing the rule (validation), iii) assessing the impact of the rule on physician behavior and clinical outcomes (impact analysis). Appropriate validation requires a prospective assessment of rule performances in multiple clinical settings, aside from the derivation study. The French Emergency Medicine Society (SFMU) and the Francophone Group of Pediatric Resuscitation and Emergency (GFRUP) have been recommending the PECARN rule since 2012 for the management of MHT in children [14, 15]. The authors emphasized the need, however, to conduct an external validation study of this CDR.

In 2014, a two-center cross-sectional study aimed to externally validate the PECARN rule [16]. The sensitivity was similar to the PECARN validation study: 100 %, 95 % CI 83.2–100, but the study had limitations that prevented the CDR being rigorously validated. Thus, two-thirds of the patients were enrolled in a pediatric emergency department located in USA, as in the derivation study. Part of the study was retrospective, however, and no clinical follow-up was performed for all of the patients.

Since the PECARN rule has not been externally validated to date in regard to methodological standards, we performed a multicenter prospective cohort study of children presenting to the emergency department (ED) with MHT. Our goal was to evaluate the diagnostic performance of the PECARN rule for identifying ciTBI in a French pediatric population.

## Methods

### Study design and settings

We conducted a multicenter, prospective, non-interventional cohort study of patients with minor head trauma who presented to three emergency departments (EDs) in France. We enrolled the patients from May of 2013 to May of 2014 in the pediatric ED of the Nantes University Hospital (35,000 visits per year), and from June 2014 to October 2015 in the EDs of two general hospitals (GH), located in Saint-Nazaire (GH1: pediatric ED with 12,500 visits per year) and La Roche-sur-Yon (GH2: general ED with 7200 pediatric visits per year).

In keeping with French legislation and ethics guidelines, non-opposition to research participation was obtained from legal representatives after providing oral and written information. The study was approved by the National Commission of Data Protection and Liberties (CNIL).

### Participating patients

We included children less than 16 years of age who presented to the ED within the 24 h after a blunt head trauma with an initial GCS ≥14.

In keeping with the PECARN study, we excluded patients with GCS score of less than 14, patients with trivial injury mechanisms (ground level falls, walking into stationary objects, and no signs or symptoms of head trauma other than scalp abrasions or lacerations) and patients who had received a CT scan prior to the ED consultation. We also excluded patients with penetrating trauma, pre-existing neurologic disorders including brain tumors, or bleeding disorders. We assessed enrolment bias by identifying non-enrolled eligible patients through review of the ED patient logs.

### Data collection

Pediatricians, emergency medicine physicians, or residents prospectively completed all of the study forms when the children were first examined in the ED. All clinical and radiological characteristics were recorded, and the physicians checked whether there were predictor variables associated with a risk of clinically-important traumatic brain injury (ciTBI).

**Fig. 1:**
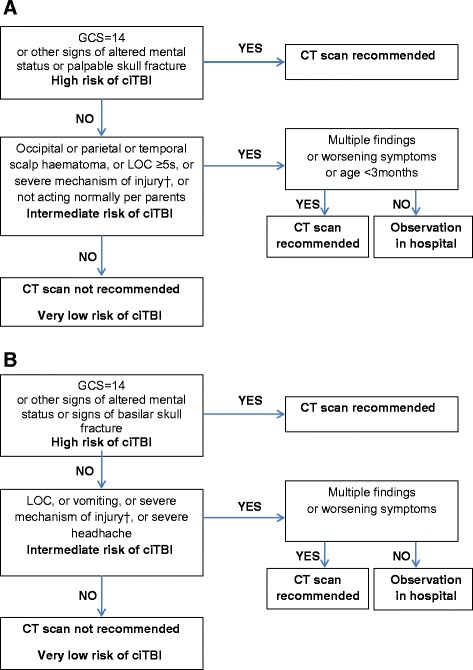
PECARN rules for children: (**a**) younger than 2 years, (**b**) aged 2 years and older. *GCS* Glasgow Coma Scale, *ciTBI* clinically-important traumatic brain injury, *CT* computed tomography, *LOC* loss of consciousness. † Severe mechanism of injury: motor vehicle crash with patient ejection, death of another passenger, or rollover; pedestrian or bicyclist without helmet struck by a motorized vehicle; fall of more than 0.9 m for children younger than 2 years, or more than 1.5 m for children aged 2 years and older; or head struck by a high-impact object

The study populations were stratified into three groups at risk for ciTBI according to the PECARN rules (i.e. very low, intermediate, and high-risk) (Fig. 1). Children in the high-risk group received a CT scan. Children in the intermediate risk group were placed under observation in the hospital, and they had a CT scan if they had multiple predictors of ciTBI, if their symptoms deteriorated during the period of observation, or if they were less than 3 months old. For the very low-risk group, children were discharged from the ED without receiving a CT scan or hospitalization. Parents of children who left the hospital were advised in writing that they should monitor their child’s wellbeing over the next 48 h.

To identify missed TBIs, parents of children who were discharged from the ED were contacted by telephone from 30 to 90 days after the hospital visit for the head trauma, using a standardized interview. The aim was to identify patients who had received any neuroimaging or had needed any secondary clinical interventions for the management of their head injuries. If there was any indication during the follow-up period that a TBI had been missed, clinical and medical records were obtained. If the physician was not able to get in contact with the family, the medical and the county morgue records were reviewed in the hospital where the patient was admitted, in order to detect a subsequently diagnosis of ciTBI. For patients included in Nantes Hospital and unreachable, we also consulted the medical registers in the two nearby hospitals with a pediatric neurosurgery service (Rennes and Angers University Hospitals).

### Outcome measures

Our main outcome was the occurrence of a ciTBI defined as death, neurosurgery, intubation induced due to the TBI for more than 24 h, or a hospital admission of two nights or more associated with a TBI seen on CT.

CT scans were performed with helicoidal CT scanners, with radiographic slices separated by 5 mm or less. CT scans were interpreted onsite by radiologists, and TBI as seen on CT was defined by the presence of any of the following criteria: diastasis of the skull and/or skull fracture, pneumocephalus, intracranial hemorrhage or contusion, sigmoid sinus thrombosis, traumatic infarction, diffuse axonal injury or signs of herniation.

### Statistical analysis

We described the data with population proportions with 95 % confidence intervals (CI). We calculated the performance of the PECARN TBI rules for the outcome measure (ciTBI). We reported these analyses for the age-based rules independently and combined together. Sensitivity, specificity, as well as positive and negative predictive values were determined after a contingency table was generated. We calculated the positive and negative likelihood ratios. The post-test probabilities were determined using a Fagan Nomogram.

## Results

Between May of 2013 and October of 2015, we enrolled 1595 (60 %) of the 2644 children with minor head trauma who presented to the ED. Characteristics between enrolled and non-enrolled patients were different in terms of the mean age, which was 4.4 vs. 5 years (*p* <0.0001), respectively; and gender, which was 64 % vs. 59 % males (*p* = 0.005), respectively; and they were similar in terms of CT rates, which were 5.1 % vs. 5.8 % (*p* = 0.44), respectively; and ciTBI rates, which were 0.6 % vs. 1.3 % (*p* = 0.06), respectively. The majority of missed patients presented to the ED during overnight hours, when there were fewer physicians on duty and they did not have time to complete data forms.

**Fig. 2:**
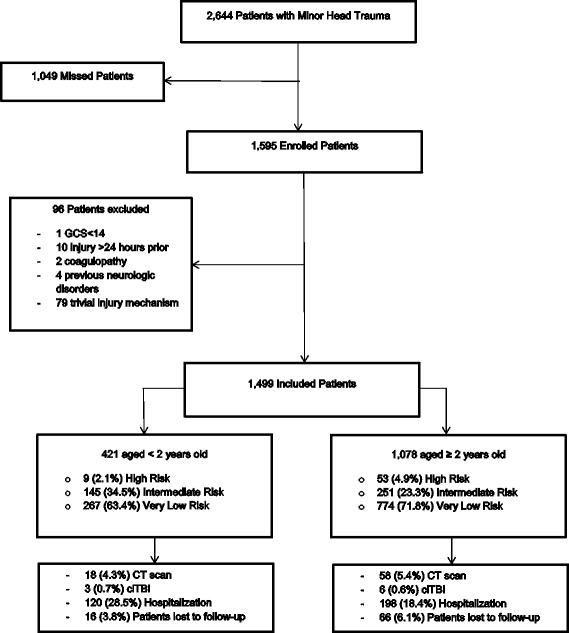
Flow chart. *GCS* Glasgow Coma Scale, *ciTBI* clinically-important traumatic brain injury, *CT* computed tomography

We excluded 96 enrolled patients because they presented greater than 24 h after the injury or with trivial injury mechanisms (defined by ground-level falls or walking into stationary objects, and no signs or symptoms of head trauma other than scalp abrasion). We also excluded one patient with a GCS under 14, two patients with coagulopathy, and four with previous neurological disorders (Fig. 2).

Of the 1499 included children, 373 (25 %) of these patients were assessed in GH1, 239 (16 %) children in GH2 (representing a total of 41 % patients in general hospitals) and 887 (59 %) patients in the Nantes University Hospital. The median age was 3 years (interquartile range, 1.7–6). 421 (28 %) patients were under 2 years of age, and 955 (64 %) were male. The sex ratio was 1.8. Clinical characteristics of the study patients, as compared to the PECARN original validation study, are presented in Table 1. Nearly all study patients (98.5 %) had a GCS of 15.

**Table 1:** Patient characteristics in the study cohort compared with the validation cohort of PECARN [2]

Characteristics	Study cohort, *n* = 1499 *n* (%)	PECARN, *n* = 8627 *n* (%)
Median age (IQR), y	3 (1.7–6)	7.1^a^
Male	955 (64)	NR
<2 years of age	421 (28)	2216 (25.7)
Risk of ciTBI		
*High risk*	62 (4.1)	1468 (17) *
GCS score = 14	23 (1.5)	255 (3) *
Altered Mental Status	47 (3.1)	1082 (12.6) *
Signs of basilar skull fracture	26 (2.4)	51 (0.8) *
Palpable skull fracture	3 (0.7)	80 (3.6) *
*Intermediate risk*	396 (26.4)	2183 (25.3)
Severe mechanism of injury	251 (16.7)	1271 (14.9)
Non frontal haematoma	47 (11.2)	361 (16.5) *
Loss of consciousness	91 (6.1)	1160 (14.1) *
Vomiting	183 (17)	1050 (12.3) *
Severe headache	15 (1.4)	146 (2.8) *
Not acting normally	29 (6.9)	273 (12.7) *
*Very low risk*	1041 (69.5)	4976 (57.7) *
CT	76 (5.1)	2917 (33.8) *
Any injury on CT	20 (1.3)	184 (6.3) *
ciTBI	9 (0.6)	88 (1)
Neurosurgery	0 (0)	16 (0.2)

*IQR* interquartile range, *y* years, *NR* not reported, *ciTBI* clinically-important traumatic brain injury, *CT* computed tomography

^a^Mean age in derivation and validation cohort; * *p*-value <0.05

A CT scan was performed on 76 patients (5.1 %), while no MRI was performed. Of the 1499 included patients, nine children (0.6 %; 95 % CI 0.3 to 1.1 %) had a clinically-important traumatic brain injury. All nine had been under observation for at least two nights in a hospital in light of their symptoms. None of them underwent neurosurgery, required intubation for more than 24 h, or died from their injury. Among all of the patients who received a CT scan, 11 had an isolated skull fracture without intra-cranial hemorrhaging; six were under 2 years of age (54 %). They all had been hospitalized for observation and none of them needed neurosurgery. The number of CT scans performed to identify a single ciTBI was eight. The CT rates differed between participating centers: 3/373 (0.8 %) in GH1, vs. 17/239 (7 %) in GH2 (*p* <0.0001). While the ciTBI rates (0 % in these two GH) were similar, the proportion of patients in the high-risk group differed considerably: 6/373 (1.6 %) in GH1 vs. 20/239 (8.4 %) in GH2 (*p* <0.0001). The CT rate in the Nantes hospital was 56/887 (6.3 %) and all of the patients with ciTBI presented to this pediatric ED. The CT rates varied by risk groups: 25/62 (40 %) of high-risk patients, 47/396 (11.9 %) of intermediate-risk patients and 4/1041 (0.4 %) of very low-risk patients had a CT scan (*p* <0.0001). 318 (21.2 %) children were hospitalized for observation, and none needed neuro-imaging upon deterioration of their symptoms.

Of the 1499 included patients, 1282 (85.5 %) were managed according to the PECARN age-based TBI clinical prediction rules. Among the remaining 217 patients (14.5 %), 85 % were managed following a more mild medical strategy in comparison to the PECARN rule (observation rather than CT scan or return home rather than hospitalization) and the other 15 % received medical care by excess (CT scan or hospitalization which was not recommended by the rule). The outcome measure was determined for 1417/1499 (95 %) of the patients through telephone follow-ups or CT scans. We completed clinical follow-ups for 1341 of the 1423 (94 %) patients who did not have neuro-imaging, through telephone interviews with the patients’ guardians. For the 82 patients who were not contacted by telephone, medical hospital records were reviewed, as were morgue records. No patient discharged from the ED underwent a CT scan in the original hospital for the same head trauma. Of the 26 patients included in Nantes University Hospital and lost to follow-up, none had been hospitalized or needed neurosurgery in the two nearby hospitals with a pediatric neurosurgery service. No patient with ciTBI had been missed according to our follow-up. We assessed the overall diagnostic accuracy of the PECARN rule, as well as for children <2 years of age and ≥2 years, for detection of ciTBI (Table 2). No patient with ciTBI was misclassified in the very low-risk group, two were classified in the intermediate-risk group, and seven in the high-risk group.

**Table 2:** Performance of the PECARN clinical decision rules by age and combinated

	ciTBI		Sensitivity	Specificity	Negative predictive value	Positive predictive value
PECARN ciTBI risk group	Yes	No	% (95 % CI)	% (95 % CI)	% (95 % CI)	% (95 % CI)
Children <2 years						
Intermediate or high risk	3	151	100 (29–100)	64 (59–69)	100 (99–100)	2 (0–6)
Very low risk	0	267				
Children ≥2 years						
Intermediate or high risk	6	298	100 (54–100)	72 (69–75)	100 (99–100)	2 (1–4)
Very low risk	0	774				
Overall						
Intermediate or high risk	9	449	100 (66–100)	70 (68–72)	100 (99–100)	2 (1–4)
Very low risk	0	1041				

*ciTBI* clinically-important traumatic brain injury, *PECARN* Pediatric Emergency Care Applied Research Network, *CI* confidence interval

**Fig. 3:**
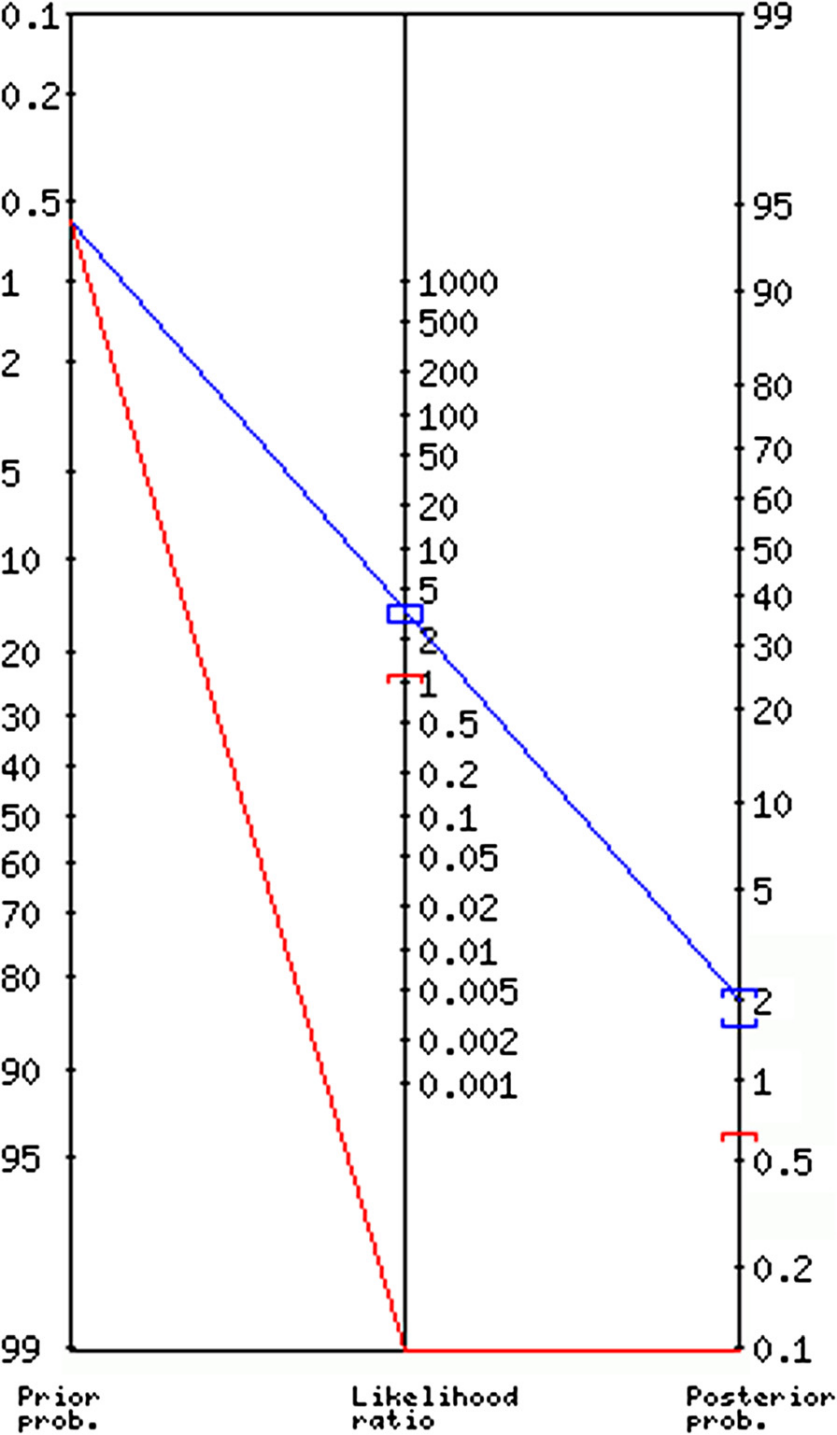
Fagan nomogram of the PECARN rule. The pre-test probability (prevalence) was 0.6 % (95 % CI 0.27 to 1.14 %) and the post-test probability was 0 % (95 % CI 0 to 1 %) when the rule was negative

In our study, the positive likelihood ratio was 3.3 (95 % CI 3.1 to 3.6), and the negative likelihood ratio was 0 (95 % CI 0 to 1.1). The Fagan nomogram for the PECARN rule can be found in Fig. 3.

## Discussion

Our study in a French pediatric population confirmed the good predictive performances of the PECARN clinical decision rule for minor head trauma in children, with a sensitivity of 100 % (95 % CI 66.4 to 100 %) and a negative predictive value of 100 % (95 % CI 99.7 to 100 %). The PECARN rule performed similarly to our study, to its internal validation study, and to the only other published external validation study [2, 16]. This prediction rule reliably successfully identified children at very low-risk of ciTBI who can safely return home without undergoing a CT scan.

Our external validation study in a cohort of 1499 children with minor head trauma was the first one according to the methodological standards published by the Evidence-Based Medicine Working Group in 2000 [13]. Our study was conducted prospectively in a completely new population, in new multiple clinical settings, by clinicians who were not the same ones involved in the derivation study. It is the only validation study undertaken in academic pediatric EDs, non-academic pediatric EDs, and general EDs, while also employing a wide variety of physicians and not only pediatric emergency subspecialists. Our results might apply generally to non-academic EDs that provide care for children. Furthermore, clinical follow-up was performed for almost 95 % of the patients discharged from the ED without a CT scan; while for the others, medical records were reviewed to identify repeat visits. In our study, no child needed a CT scan secondarily, and no ciTBI was missed.

Our study has some limitations however. First, we did not enroll all of the patients, thus potentially leading to a selection bias. When enrolled and missed patients were compared, differences in the median age and in the percentage of patients younger than 2 years of age were found, although we could not discern any differences between enrolled and non-enrolled patients in terms of their CT rates and their ciTBI rates. Based on a review of the medical records of non-enrolled patients, it appeared that most were non-enrolled because their injuries were trivial, and hence did not entail a risk of TBI. Enrolled patients constituted a representative spectrum of the severities of minor head traumas. Second, for two thirds of patients lost to follow-up, medical records were reviewed only in the original hospital, which could lead to missing hospitalizations or CT scans in other neighboring centers. Last, we also identified only nine clinically-important traumatic brain injuries, due to its low prevalence in children with minor head trauma (0.6 % in our study, 0.9 % in PECARN study). This limited the accuracy of the rule sensitivity.

CDRs for children with MHT are needed because head trauma is very common and CT use is increasing [5]. This leads to exposure of a large number of children to ionizing radiation from CT, which is associated with an increased lifetime risk for malignancies [6, 7]. The small risk of ciTBI after MHT should be compared with the risks derived from ionizing radiation exposure with CT scans; especially in children younger than 2 years of age, as they are the most sensitive to radiation. The PECARN rule appears the best for children and infants with the largest cohort and highest sensitivity for detection of ciTBI, but some authors fear that its application would result in an excessive increase in the rate of CT use [17, 18]. In our study, the CT rate was 5.1 % and the rate of CT scans per ciTBI was 8 %. These values are lower than those found in the PECARN validation study and in the others CDR derivated in children with MHT [2, 3, 19]. In our population, the PECARN rule seems to meet the objective of limiting the use of CT, without ignoring brain injuries. The potential reduction in CT use by application of this rule could be greater in general hospitals where the rates of CT use with children are higher than in pediatric EDs [20, 21]. The need to conduct a validation study in a new population was confirmed by the results of our study: significant differences between the derivation population and our French study population were encountered. More children with high-risk predictors were admitted in EDs in the USA than in French EDs, leading to higher scan rates in the PECARN study.

More than 85 % of our patients had been managed according to the PECARN rules. The implementation of a clinical decision rule, even if it has been shown to be valid and reliable is not always easy. A high level of scientific evidence is not always enough to make a rule applicable in everyday life, and to entice physicians to change their habits. Several factors will determine the success of the implementation of the rule. These are: the physician’s clinical experience, the potential medicolegal consequences, and the patient’s request. The latter is particularly the case in pediatric EDs due to the frequent parental preference for an examination. In this case, the rule could help physicians with informing parents of the different management strategies that depend on ciTBI risks versus the risks associated with CT use. A CDR is always assistive rather than directive, and it is meant to help clinicians, not to replace their decision making.

## Conclusions

We conducted a prospective multicenter validation study of the PECARN clinical decision rule for detection of ciTBI in children with minor head trauma, according to the methodological standards. The PECARN rule successfully identified all of the patients with ciTBI, with a limited use of CT scans. A broad validation study with a large cohort is needed to allow sufficient statistical power before authorizing its implementation and generalization. Such a study is currently underway, with recruitment taking place in nine French general and pediatric EDs (ClinicalTrials.gov Identifier: NCT02357186).

## Abbreviations

95 % CI, 95 % confidence interval; CDR, clinical decision rule; ciTBI, clinically-important traumatic brain injury; CT, cranial computed tomography; ED, emergency department; GCS, Glasgow coma scale; GFRUP, Francophone Group of Pediatric Resuscitation and Emergency; GH, General Hospital; MHT, minor head trauma; PECARN, Pediatric Emergency Care Applied Research Network; SFMU, French Emergency Medicine Society; TBI, traumatic brain injury
